# Biomass-Derived Oxygen and Nitrogen Co-Doped Porous Carbon with Hierarchical Architecture as Sulfur Hosts for High-Performance Lithium/Sulfur Batteries

**DOI:** 10.3390/nano7110402

**Published:** 2017-11-21

**Authors:** Yan Zhao, Li Wang, Lanyan Huang, Maxim. Yu. Maximov, Mingliang Jin, Yongguang Zhang, Xin Wang, Guofu Zhou

**Affiliations:** 1 International Academy of Optoelectronics at Zhaoqing, South China Normal University, Zhaoqing 526060; China; zhaoyan84830@hotmail.com (Y.Z.); lanyan.huang@zq-scnu.org (L.H.); jinml@scnu.edu.cn (M.J.); guofu.zhou@m.scnu.edu.cn (G.Z.); 2 Synergy Innovation Institute of GDUT, Heyuan 517000, China; 3 Peter the Great Saint-Petersburg Polytechnic University, Saint-Petersburg 195221, Russia; maximspbstu@mail.ru; 4 Institute of Electronic Paper Displays, South China Academy of Advanced Optoelectronics, South China Normal University, Guangzhou 510631, China; li.wang@guohua-oet.com

**Keywords:** lithium/sulfur battery, composite cathode, hierarchically porous carbon, oxygen and nitrogen co-doping

## Abstract

In this work, a facile strategy to synthesize oxygen and nitrogen co-doped porous carbon (ONPC) is reported by one-step pyrolysis of waste coffee grounds. As-prepared ONPC possesses highly rich micro/mesopores as well as abundant oxygen and nitrogen co-doping, which is applied to sulfur hosts as lithium/sulfur batteries’ appropriate cathodes. In battery testing, the sulfur/oxygen and nitrogen co-doped porous carbon (S/ONPC) composite materials reveal a high initial capacity of 1150 mAh·g^−1^ as well as a reversible capacity of 613 mAh·g^−1^ after the 100th cycle at 0.2 C. Furthermore, when current density increases to 1 C, a discharge capacity of 331 mAh·g^−1^ is still attainable. Due to the hierarchical porous framework and oxygen/nitrogen co-doping, the S/ONPC composite exhibits a high utilization of sulfur and good electrochemical performance via the immobilization of the polysulfides through strong chemical binding.

## 1. Introduction

The lithium/sulfur (Li/S) battery system holds abundant promise in the development of next-generation rechargeable batteries due to its unparalleled theoretical capacity (1672 mAh·g^−1^), high theoretical energy density (2600 Wh·kg^−1^), low cost, low environmental pollution, and natural abundance [1]. However, several issues impede the practical use of sulfur cathode materials, including the poor electron conductivity of elemental S and the high solubility of lithium polysulfides (Li_2_S*_x_*, 4 ≤ *x* ≤ 8) into the organic electrolytes, which leads to a failure for battery performance [2]. Thus, numerous studies have been conducted to enhance the electrochemical performance of Li/S batteries, mainly by immersing in sulfur with diverse conductive polymers and carbon materials, such as polypyrrole, graphene, carbon nanotubes, and porous carbon [3,4,5,6,7].

The obvious advantages of carbon materials include their high conductivity and good porosity, and thus carbon has been demonstrated as an effective sulfur immobilizer for Li/S batteries with high performance. For example, Xi et al. prepared abundant porous carbon samples from different zinc-containing metal-organic frameworks, which possessed both mesoporosity and microporosity for good sulfur loading and electrochemical utilization [8]. A porous nano-sized spherical carbon was prepared by Schuster et al. with a two-step casting process, to form the cathode of lithium/sulfur batteries in combination with sulfur via a melt-diffusion strategy. As-prepared carbon with an abundant surface area of 2445 m^2^·g^−1^ allows the sulfur to distribute homogeneously into the pores, greatly enhancing the electrochemical behavior of the corresponding cathodes [7].

However, the weak mutual effect between carbon host and sulfur, based on physical adsorption, limit the aforementioned designs. Recently, heteroatom dopants, such as oxygen and nitrogen, on carbon materials has been found to play an important role in trapping lithium polysulfides via strong chemical binding [9,10,11]. For example, Zhang et al. demonstrated that the effective chemical binding of S to the oxygen-containing group of graphene can significantly retard the dissolution/diffusion of lithium polysulfides [9,10]. Yu et al. fabricated a novel nitrogen-enriched hierarchical porous carbon via the simple thermolysis of magnesium citrate and NH_3_ to host sulfur as a Li/S battery cathode. The resulting cathode yields a large reversible capacity and retains a stable reversible capacity even after the 300th cycle of the charge/discharge process at 1 C [11].

Although the above S/C composite materials are well designed at the lab scale, they are still far from commercialization because of the relatively high cost and complicated synthetic approaches. Therefore, there is a need to focus on the preparation of low-cost carbon materials for facilely hosting sulfur. Notably, porous carbons from biomass materials have shown their potential in Li/S battery systems due to their abundance and environmental friendliness as well as the possibility of avoiding the use the sophisticated chemical procedures, in introducing heteroatom dopants into the carbon matrix [12,13,14]. Over the past decade, various biomass materials have been developed for use in supercapacitors, lithium-ion batteries, and sodium-ion batteries. Wang et al. reported a biomass-based activated carbon prepared from willow catkins and applied as electrode materials for supercapacitors with maximal specific capacitances of 340 F g^−1^ [15]. Jiang et al. synthesized biomass carbon fibers derived from corncobs and ramie fibers, showing the specific capacity of 606 and 489 mAh·g^−1^ in lithium-ion batteries, respectively [16]. Zhang et al. reported a carbon microtube synthesized via carbonizing poplar catkin. The carbon microtube was composited with sulfur, which could display the specific discharge capacity of 810 mAh·g^−1^ after 100 cycles at 0.1 C [14]. Through the above, we can find that biomass carbon materials have good application prospects. 

Spent coffee grounds is an agricultural waste with a high yield across the globe. It is a promising carbon precursor for producing low-cost activated carbon, and its application in anodes for lithium-ion batteries has been reported in previous study [17]. 

To the best of our knowledge, little attention has been paid to the feasibility of porous carbon derived from waste coffee grounds to composite with sulfur for Li/S battery cathode application. In this study, waste coffee grounds was selected as a precursor to prepare oxygen and nitrogen co-doped porous carbon (ONPC) as a conducting matrix to host sulfur for the fabrication of cathodes for Li/S batteries. The effects of pore structure and oxygen/nitrogen co-doping on the electrochemical performance of the S/ONPC composite cathodes were investigated systematically.

## 2. Materials and Methods

The ONPC was prepared by the thermal carbonization of waste coffee grounds. Firstly, waste coffee grounds were ultrasonically cleaned with deionized (DI) water several times and dried at 60 °C under vacuum for 12 h. Then the mixture of the waste coffee grounds and ZnCl_2_ in the weight ratio of 1:1 was wet ball-milled with a small quantity of DI water at 200 rpm for 3 h. The obtained paste mixture was thoroughly dried at 90 °C for 12 h. After the water had evaporated, the sample was carbonized at 900 °C for 3 h under an Ar flow. Finally, the black ONPC was produced after washing by 0.6 M HCl and DI water alternately and drying in vacuum at 60 °C. The S/ONPC composite was prepared via a solvothermal synthesis method [18]. Firstly, 0.4 g of ONPC powder was ultrasonically dispersed in 1.0 g of S/CS_2_ solution with 50 wt % S for 2 h. After drying, the resulting black sample was placed in a ceramic boat and calcined at 150 °C for 10 h in a N_2_ flow to obtain the S/ONPC composite. 

X-ray diffraction (XRD) was used to analyze the crystalline structure of the samples via D8 advance (Bruker, Karlsruhe, Germany) with Cu Kα radiation. Scanning electron microscopy (SEM) and transmission electron microscopy (TEM) images were obtained on a scanning electron microscope (SEM, S4800, Hitachi Limited, Tokyo, Japan) and transmission electron microscope (TEM, JEM-2100F, JEOL, Tokyo, Japan). Dispersive Spectrometer (EDS) mapping was performed by TEM at 160 kV to analyze the elements distribution. X-ray photoelectron spectroscopy (XPS) was utilized to analyze the chemical composition via PHI 5000C ESCA System (Ulvac-Phi, Kanagawa, Japan). The nitrogen adsorption/desorption isotherms were measured via Quabrasorb, SI-3MP (Quantachrome Instrument, Boynton Beach, FL, USA). Thermogravimetric analysis (TGA, SDT Q-600, TA Instruments-Waters LLC, Newcastle, PA, USA) was carried out from 25 to 700 °C with a heating rate of 10 °C·min^−1^.

For sulfur electrodes, the S/ONPC composite was mixed with acetylene black and polyvinylidene fluoride (PVDF) binder dissolved in *N*-methyl-2-pyrrolidinone (NMP) solution with a mass ratio of 8:1:1 to obtain a homogeneous slurry. The above slurry was spread on an Al foil current collector and subsequently dried in vacuum at 60 °C for 12 h. Before assembling the cells, the sulfur cathode was cut into disks with a diameter of 12 mm. The active materials loading on each disk was controlled to 2–2.5 mg. The electrolyte was 1 M lithium trifluoromethanesulfonate (LiCF_3_SO_4_) in a mixed solution of dimethoxy ethane and 1,3-dioxolane (1:1, *v*/*v*). The coin cells (CR2025) were assembled in an Ar-filled glove box (MBraun, Garching, Germany) and aged for 12 h before battery tests. A battery test instrument (LAND CT2001A, Shenglan, Wuhan, China) was used for investigating the cell cyclability at different current densities in the voltage range of 1–3 V vs. Li^+^/Li. The Versa STAT electrochemical workstation (Princeton, VersaSTAT 4, 50/60 Hz, Ametek, PA, USA) was used to test electrochemical impedance spectroscopy (EIS) in the frequency range of 0.01–100 kHz. 

## 3. Results and Discussion

**Figure 1:**
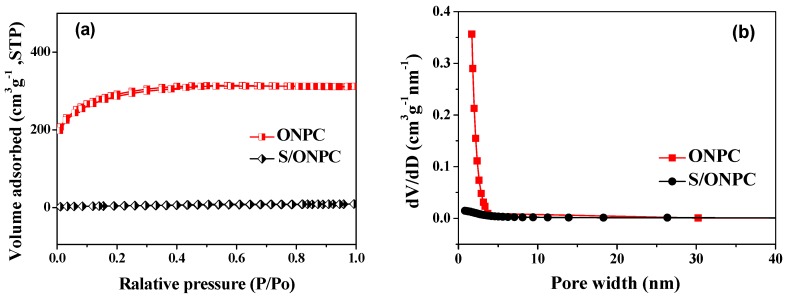
Nitrogen adsorption/desorption isotherms (**a**) and size distribution (**b**) of the ONPC and S/ONPC composite.

Figure 1a reveals the nitrogen adsorption/desorption isotherms of the ONPC and the S/ONPC composite. The ONPC exhibits typical type I isotherms with unapparent hysteresis loop, indicating the micro/mesopore structure of the ONPC [15,19]. Figure 1b presents the pore size distribution (PSD) curves of the ONPC and the S/ONPC composite. From the PSD curve of the ONPC, the micro/mesopore structure is also evident. The Brunauer-Emmett-Teller (BET) surface areas, pore volumes, and the micropore surface areas of the ONPC and the S/ONPC composite are summarized in Table 1. As listed in Table 1, the surface area of ONPC was 1017.5 m^2^·g^−1^ with the pore volume of 0.48 cm^3^·g^−1^. After the impregnation of sulfur, the surface area of the S/ONPC composite was only 22.4 m^2^·g^−1^ with the pore volume of 0.015 cm^3^·g^−1^. The significant reduction in surface area and pore volume is mainly ascribed to the encapsulation of a large amount of sulfur in ONPC. In general, the ONPC possesses a large pore volume and abundant specific surface area, which could effectively retard the diffusion of polysulfides and diminish the shuttle effect in the charge/discharge process of an Li/S battery [20,21].

**Figure 2:**
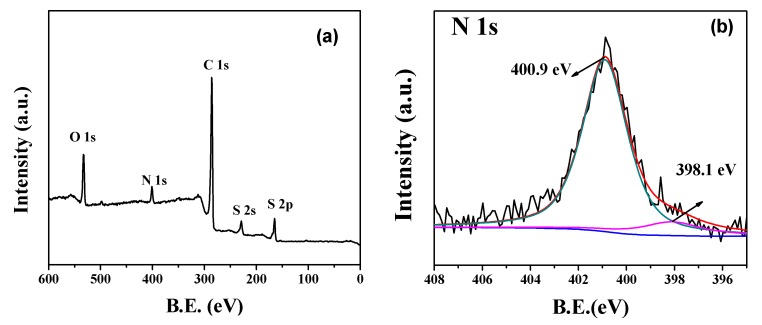

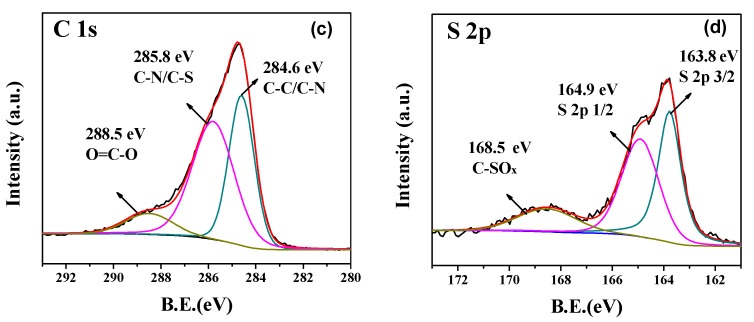
(**a**) XPS spectra of S/ONPC composite, (**b**) N1s XPS spectrum of the S/ONPC composite, (**c**) C1s XPS spectrum of the S/ONPC composite, (**d**) S2p XPS spectrum of the S/ONPC composite.

The XPS spectra were used to determine the surface chemistry of the S/ONPC composite. The XPS survey spectra of S/ONPC (Figure 2a) shows five peaks centered at 163.6, 227.6, 286.4, 401.5, and 532.8 eV, which can be assigned to S2p, S2s, C1s, N1s, and O1s, respectively. As shown in high-resolution N1s spectra (Figure 2b), the N1s spectrum can be fitted into two individual peaks with binding energies of 400.9 and 398.1 eV, corresponding to graphitic-*N* and pyridinic-*N* [22]. It is reported that the doped nitrogen may enable an improvement of the electric conductibility, the wettability in the electrolytes, and the adsorption of polysulfides on the carbon matrix [11,23]. As for the C1s spectrum of the S/ONPC composite (Figure 2c), three peaks at 288.5, 285.8, and 284.6 eV can be associated to O–C=O, C–N/C–S, and C–C/C–N bonding, suggesting that oxygen-functional groups exist in the S/ONPC composite [24]. According to the report, the existence of oxygen-containing functional groups can favor the binding ability of lithium polysulfides and improve the utilization of active materials, leading to a good cycling ability for Li/S batteries [25]. The S2p binding energy peak of the S/ONPC composite can be separated into three peaks, including the S2p 3/2 peak (163.8 eV), S2p 1/2 peak (164.9 eV), and C–SO*_x_* (168.5 eV), as shown in Figure 2d [26]. The XPS results show that the S atoms are chemically bonded to the ONPC, which could effectively immobilize sulfur, inhibit the diffusion of soluble polysulfides, and thus improve the electrochemical performance of the S/ONPC composite.

**Figure 3:**
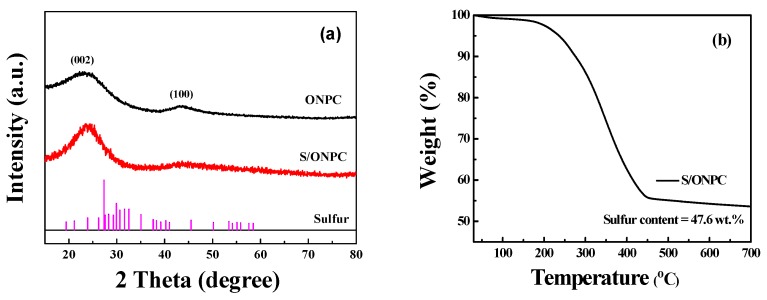
XRD patterns of the ONPC and the S/ONPC composite (**a**); Thermogravimetric (TG) curve of the S/ONPC composite (**b**).

Figure 3a shows the XRD patterns of the as-prepared ONPC and the S/ONPC composite. It can be seen that the ONPC demonstrates two broad peaks located at 23.4° and 43.4°, which are assigned to the (002) and (100) planes of the graphite lattice [27]. The partially graphitized structure can improve the conductivity of the ONPC, thereby favoring the rate ability of the S/ONPC [28]. The S/ONPC composite exhibits a similar XRD pattern to ONPC with no obvious crystalline sulfur peak (JCPDS No. 08-0247) [29], suggesting that the sulfur has an amorphous phase and is homogeneously distributed in the internal pores of ONPC [29]. To illustrate the actual content of sulfur, the thermogravimetric analysis (TGA) of the S/ONPC was carried out under a nitrogen atmosphere. Figure 3b shows the TG curve of the S/ONPC composite. It can be seen that the S/ONPC delivers a significant weight loss between 200 and 450 °C due to the evaporation of sulfur, and the actual sulfur content is calculated to be 47.6 wt %. The temperature at which sulfur evaporates is higher than that of the nitrogen undoped carbon/sulfur composites (200–330 °C) reported in previous studies [30], which may arise from the great encapsulation capability of nanoporous properties and the strong interaction between the sulfur and the oxygen/nitrogen atoms.

**Figure 4:**
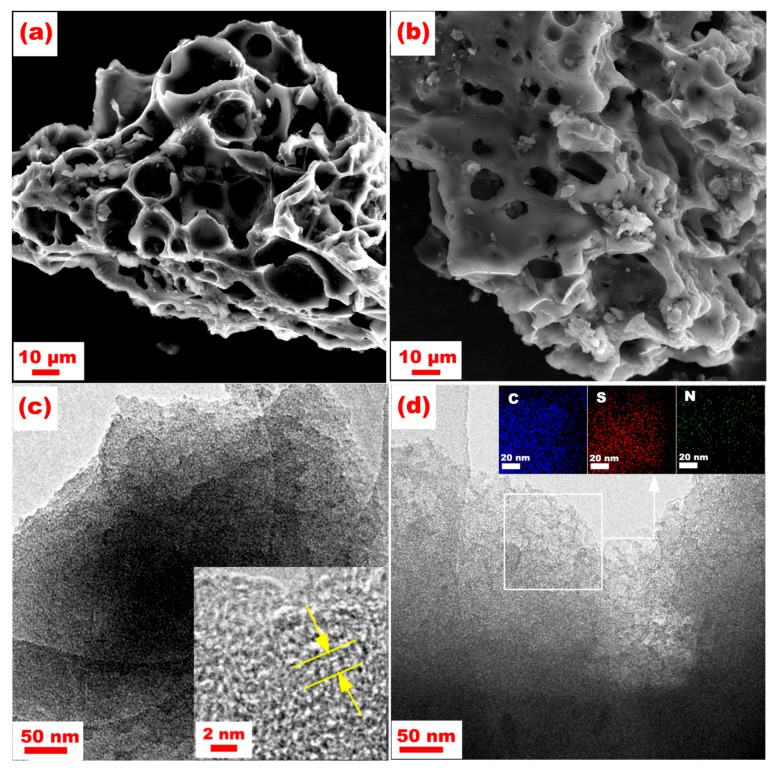
SEM images of ONPC (**a**) and S/ONPC (**b**), and TEM images of ONPC (**c**) and S/ONPC (**d**) including their high magnified views.

Figure 4a shows an SEM image of the ONPC with a three-dimensional (3D) multi-channel porous architecture. The interconnected pores of several micrometers are still maintained after hosting sulfur on the ONPC (Figure 4b), which is kinetically favorable for Li-ion/electron transportation contributing to remarkable rate capability as well as cyclability. Figure 4c presents a TEM image of the ONPC, where a meso/microporous structure is evident. These mesopores and micropores are in favor of the uniform dispersion of sulfur. Specifically, amorphous carbon containing partially graphitized carbon can be evidenced by the inset of Figure 4c of the HR-TEM image, which corresponds to the XRD results. Figure 4d reveals the TEM image of the S/ONPC composite, and no crystal sulfur can be observed, indicating the well-embedded sulfur in the composite. The Energy Dispersive Spectrometer (EDS) elemental mapping based on a selected area of the TEM image of the S/ONPC can further demonstrate that the sulfur is homogeneously distributed in the composite. In addition, some mesopores still exist after loading sulfur on the ONPC, serving as the transport pathway for Li-ion/electron and withstanding the volume expansion of sulfur during cycling.

**Figure 5:**
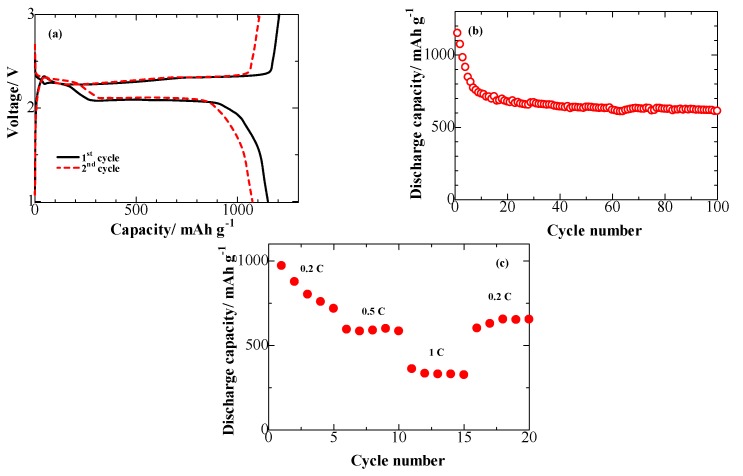
(**a**) The galvanostatic discharge/charge profiles of a lithium cell with the S/ONPC composite, (**b**) Cycle performance of a lithium cell with the S/ONPC composite for 100 cycles at 0.2 C, (**c**) Rate capability of a lithium cell with the S/ONPC composite.

Figure 5a displays the charge/discharge profiles of the S/ONPC cathode at a current destiny of 0.2 C. The two evident discharge plateaus were observed around 2.4 and 2.0 V in the first discharge curve, which are associated with the reduction mechanisms of S_8_ to soluble long-chain lithium polysulfide (Li_2_S*_n_*, 4 ≤ *n* ≤ 8) and the decomposition of the lithium polysulfide to form insoluble short-chain lithium polysulfide (Li_2_S*_n_*, *n* ˂ 4) [30]. The charge plateaus at 2.4 V in the subsequent charge process, which is associated with the oxidation transformation of Li_2_S/Li_2_S_2_. The first discharge and charge capacities of the S/ONPC composite are 1150 and 1206 mAh·g^−1^, with an initial coulombic efficiency of 95%. Meanwhile, the second cycles deliver about 1073 mAh·g^−1^ with no obvious capacity fading, suggesting that the O/N co-doped hierarchical porous carbon frame can actually restrain the dissolution of the lithium polysulfides and minimize the shuttling loss. The S/ONPC composite exhibits a good cycling ability when tested as a cathode against lithium metal at a current destiny of 0.2 C (Figure 5b). After 100 charge/discharge processes, the reversible capacity remains as high as 613 mAh·g^−1^ with a capacity retention of 57% compare with its second cycle capacity. To evaluated the rate ability, the S/ONPC composite was tested at different current densities ranging from 0.2 to 1 C under the voltage range of 1–3 V. The trend of the rate capacity can be seen in the Figure 5c; the S/ONPC composite cathode delivers a good capacity retention of 84% after 15 cycles when the current density is restored to 0.2 C after high rate cycling, indicating the structural stability of the S/ONPC composite.

**Figure 6:**
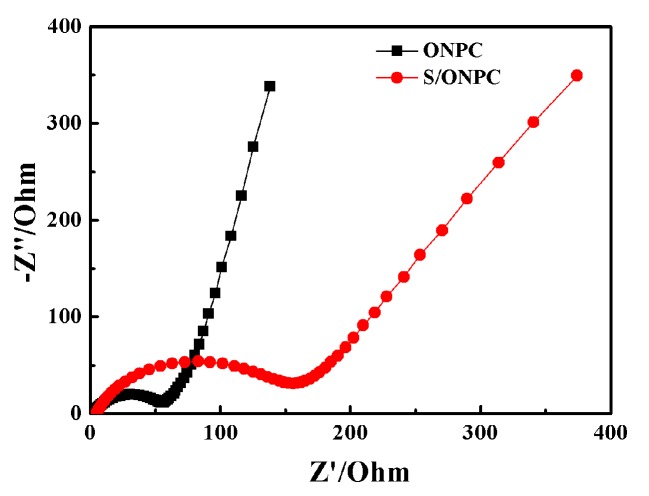
EIS results of ONPC and S/ONPC electrodes.

The EIS results of the ONPC and S/ONPC electrodes measured with an amplitude of 10 mV are displayed in Figure 6. The EIS test is able to determine the electrical conductivity of the two electrodes. The Nyquist plots of ONPC and S/ONPC electrodes both consist of typical compressed semicircles attributed to the charge transfer impedance at the high-to-medium frequency region and a sloping line in the low frequency range, which is associated with the Warburg diffusion component [3]. It can be observed that the diameter of the compressed semicircle in the high-to-medium frequency range for the ONPC electrode is smaller than that of the S/ONPC electrode. The impedance of S/ONPC is larger because the conductivity of sulfur is poor. However, the charge transfer resistance of S/ONPC is only 155 Ω, indicating that the network conductive structure of ONPC can make S/ONPC have excellent electrical conductivity.

The excellent cycling performance and rate ability of the S/ONPC composite can be related to the good conductivity of the O/N co-doped carbon frame, the adequate sulfur content, and the hierarchical porous structure. Firstly, the good conductivity of ONPC can support the fast transport of electrons into the sulfur or polysulfides and enhance the active materials utilization in the cathode. Secondly, the existence of the O/N-doped functional groups results in the good interfacial adhesion between the active materials and the electrolyte, and restrains active sulfur material loss. Moreover, the hierarchical porous structure of the S/ONPC composite provides moderate to large pore volume and specific surface area, which suppress the composite interface damages and the abuse of volume change during the cycling process.

## 4. Conclusions

In summary, oxygen and nitrogen co-doped porous carbon (ONPC) has been successfully synthesized from a direct carbonization procedure by using “zero cost” waste coffee grounds as precursors. The as-prepared carbon possesses an abundant surface area of 1017.5 m^2^·g^−1^, a high pore volume of 0.48 cm^3^·g^−1^, and a meso-/microporous structure, which is appraised as the cathode scaffolds for Li/S batteries. We have demonstrated the effect of porosity and oxygen/nitrogen doping of ONPC on the electrochemical performance of Li/S batteries. In the view of the feasible preparation approach and good electrochemical performance, the novel S/ONPC composite may become a promising cathode material in the research of Li/S batteries.

## Figures and Tables

**Table 1:** BET results (surface area and pore volume) for the ONPC and S/ONPC.

Materials	*S*_BET_ (m^2^·g^−1^)	*S*_micro_ (m^2^·g^−1^)	*V*_tot_ (cm^3^·g^−1^)	*V*_micro_ (cm^3^·g^−1^)
ONPC	1017.5	477.3	0.48	0.21
S/ONPC	22.4	3.6	0.015	0.003

*S*_BET_ = Specific surface area calculated by using the Brunauer-Emmett-Teller (BET) method. *S*_micro_ = micropore surface area calculated by the t-plot method. *V*_tot_ = total pore volume calculated by the Barrett-Joyner-Halenda (BJH) method. *V*_micro_ = micropore volume calculated by the *t*-plot method.
